# Visualization of Neuregulin 1 ectodomain shedding reveals its local processing *in vitro* and *in vivo*

**DOI:** 10.1038/srep28873

**Published:** 2016-07-01

**Authors:** Aosa Kamezaki, Fuminori Sato, Kazuhiro Aoki, Kazuhide Asakawa, Koichi Kawakami, Fumio Matsuzaki, Atsuko Sehara-Fujisawa

**Affiliations:** 1 Department of Animal Development and Physiology, Graduate School of Biostudies, Kyoto University, Kyoto 606-8501, Japan; 2 Department of Growth Regulation, Institute for Frontier Medical Sciences, Kyoto University, Kyoto 606-8507, Japan; 3 Imaging Platform for Spatio-Temporal Information, Graduate School of Medicine, Kyoto University, Kyoto 606-8501, Japan; 4 Division of Molecular and Developmental Biology, National Institute of Genetics, and Department of Genetics, SOKENDAI, Graduate University for Advanced Studies, Mishima, Shizuoka 411-8540, Japan; 5 Laboratory of Cell Asymmetry, RIKEN Center of Developmental Biology, Kobe 650-0047, Japan

## Abstract

Neuregulin1 (NRG1) plays diverse developmental roles and is likely involved in several neurological disorders including schizophrenia. The transmembrane NRG1 protein is proteolytically cleaved and released as a soluble ligand for ErbB receptors. Such post-translational processing, referred to as ‘ectodomain shedding’, is thought to be crucial for NRG1 function. However, little is known regarding the regulatory mechanism of NRG1 cleavage *in vivo*. Here, we developed a fluorescent probe, NRG1 Cleavage Indicating SenSOR (N-CISSOR), by fusing mCherry and GFP to the extracellular and intracellular domains of NRG1, respectively. N-CISSOR mimicked the subcellular localization and biochemical properties of NRG1 including cleavage dynamics and ErbB phosphorylation in cultured cells. mCherry/GFP ratio imaging of phorbol-12-myristate-13-acetate-stimulated N-CISSOR-expressing HEK293T cells enabled to monitor rapid ectodomain shedding of NRG1 at the subcellular level. Utilizing N-CISSOR in zebrafish embryos revealed preferential axonal NRG1 ectodomain shedding in developing motor neurons, demonstrating that NRG1 ectodomain shedding is spatially regulated at the subcellular level. Thus, N-CISSOR will be a valuable tool for elucidating the spatiotemporal regulation of NRG1 ectodomain shedding, both *in vitro* and *in vivo*.

Many membrane proteins are subjected to limited proteolysis, which sheds the ectodomain, a process termed “ectodomain shedding”. Such post-translational modifications of membrane proteins are critical for proper cell-cell interactions in various biological processes. However, it is not well known when and where ectodomain shedding takes place *in vivo* and how such shedding regulates intercellular signalling or adhesion.

Neuregulin 1 (NRG1), one of the major ligands of ErbB receptors, plays multiple roles in development, regeneration, synaptic plasticity, and diseases^1^,^2^,^3^. In many cases, NRG1-ErbB signalling mediates cell-cell interactions in a paracrine or juxtacrine manner. In the nervous system, NRG1 is mainly produced by neurons, while ErbB receptors are expressed in signal-recipient cells, such as immature Schwann cells and skeletal muscle cells. In addition, NRG1 expression in endocardial cells is required for the trabeculation of myocardial cells expressing ErbB2/ErbB4 during heart development^2^. The *NRG1* gene encodes multiple splicing isoforms, most of which are synthesized as transmembrane proteins and undergo proteolytic processing to release the ectodomain containing an EGF-like domain. Ectodomain shedding of NRG1 is thought to serve as a key modulator of NRG1 function, and its dysregulation is likely involved in several diseases such as schizophrenia^4^. The β-secretase 1 (BACE1)-dependent processing of NRG1 is well characterized and required for the proper myelination of Schwann cells, as well as the development and maintenance of muscle spindles, indicating that NRG1 ectodomain shedding plays critical roles in its *in vivo* functions^5^,^6^,^7^,^8^. Although the involvement of ADAM family proteases including ADAM10, ADAM17, and ADAM19 in NRG1 ectodomain shedding has been shown in cultured cells, little is known about their roles in NRG1 processing *in vivo*. The phenotypes of BACE-1-deficient mice do not completely coincide with the multiple phenotypes of NRG1 knockout mice, suggesting the potential involvement of other proteases that might include members of ADAM family.

Stimuli that induce NRG1 cleavage have been well characterized, especially in cultured cells. Phorbol-12-myristate-13-acetate (PMA), a potent activator of protein kinase C (PKC), strongly enhances NRG1 cleavage^9^,^10^. NRG1 shedding is also induced by extracellular Ca^2+^ influx and in a neuronal activity-dependent manner^11^,^12^. Activation of the p38 mitogen-activated protein kinase, a downstream effector of NRG1-ErbB signaling^13^, activates NRG1 processing^14^. In the case of neural cells, neurotrophic factors such as brain-derived neurotrophic factor (BDNF), stimulate NRG1 ectodomain shedding mediated by PKCδ^15^. Particularly noteworthy is that Schwann cell-derived BDNF stimulates the release of soluble NRG1 in axons, both *in vitro* and *in vivo*^16^,^17^. To elucidate the multiple processes leading to the axonal release of soluble NRG1, it is essential to determine when and where NRG1 is cleaved in neurons.

Here, we developed a novel fluorescent probe, designated as NRG1 Cleavage-Indicating SenSOR (N-CISSOR), to clarify when and where NRG1 is cleaved *in vitro* and *in vivo*. N-CISSOR substantially mimics the characteristics of NRG1 and enables effective monitoring of NRG1 ectodomain shedding in HEK293T cells and the motor neurons of zebrafish embryos. Our findings reveal that N-CISSOR provides a novel tool for investigating the spatiotemporal regulation of NRG1-ErbB signalling by visualizing NRG1 ectodomain shedding in single cells at subcellular resolution, both *in vitro* and *in vivo*.

## Results

### Development and characterization of the NRG1 shedding probe, N-CISSOR

**Figure 1:**
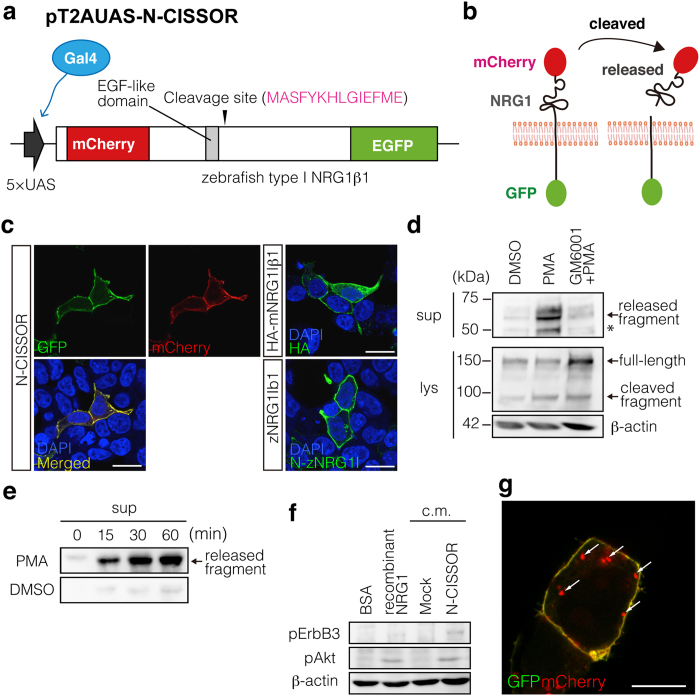
Generation of N-CISSOR probe. (**a**) N-CISSOR, type-I zebrafish *NRG1β1* (*zNRG1Iβ1*) constructed with an extracellular mCherry and an intracellular GFP, was expressed under the control of a 5× UAS sequence, which was driven by Gal4. (**b**) N-CISSOR was designed to monitor intramolecular NRG1 cleavage, which resulted in physical separation of mCherry and GFP. Ectodomain-shedding activities were assessed as relative values of the mCherry/GFP ratios. (**c**) Cellular localization of N-CISSOR expressed in HEK293T cells compared with those of immunodetected HA-mNRG1Iβ1 and unlabelled zNRG1Iβ1. Scale bar: 20 μm. (**d,e**) Western blot analysis of N-CISSOR dynamics. Released (1^st^ panel) and full-length (2^nd^ panel) N-CISSOR fragments were detected using anti-mCherry and anti-GFP antibodies, respectively. β-actin was detected as an internal control (3^rd^ panel). An asterisk shows the extra bands produced by the intramolecular cleavage of mCherry during the denaturation procedure. (**d**) Metalloprotease-dependent N-CISSOR cleavage induced by PMA treatment for 20 min. Pre- and co-treatment with GM6001 for 30 min inhibited the PMA-induced release of ectodomain fragments. (**e**) Time-dependent N-CISSOR release induced by PMA treatment. The supernatants of N-CISSOR-expressing cells were harvested at the indicated time points after PMA or DMSO treatment. (**f**) Analysis of N-CISSOR bioactivity. Differentiated C2C12 myotubes were treated with conditioned medium from N-CISSOR-expressing cells or control cells. Recombinant NRG1 and its vehicle (BSA) were used as positive and negative controls, respectively. pErbB3 (185 kDa), pAkt (60 kDa), and β-actin (60 kDa) were detected in the lysates of C2C12 myotubes. (**g**) A merged image of GFP and mCherry in a N-CISSOR-expressing cell. Intracellular mCherry dots are indicated by arrows. Scale bar: 10 μm.

We developed the fluorescent N-CISSOR probe to monitor NRG1 ectodomain shedding in living cells and zebrafish embryos. As shown in Fig. 1a,b, N-CISSOR was constructed so that mCherry and GFP were tagged onto the N-terminal and C-terminal domains of the zebrafish NRG1 protein, respectively. Cleavage of the NRG1 ectodomain within N-CISSOR released the mCherry-fused extracellular domain (ECD) from the GFP-fused intracellular domain (ICD). N-CISSOR expression was controlled by the Gal4-UAS system to enable tissue-specific expression in zebrafish embryos^18^.

We first evaluated whether N-CISSOR mimics the subcellular localization and dynamics of NRG1 in HEK293T cells. N-CISSOR mainly localized to the cell surface in HEK293T cells, similarly to HA-tagged mouse NRG1Iβ1 (HA-mNRG1) and zebrafish NRG1Iβ1 (zNRG1lβ1) (Fig. 1c). Next, we examined the metalloprotease-dependent cleavage of N-CISSOR in cultured cells following treatment with PMA, a potent PKC activator. Previous findings have shown that PMA-induced ectodomain shedding of ErbB ligands including NRG1 is mediated by ADAM17^19^. As expected, PMA treatment for 20 min dramatically enhanced the release of mCherry-fused extracellular N-CISSOR fragments into the culture medium in association with the concomitant increase of the cleaved forms of GFP-containing C-terminal fragments in cell lysates. The release of extracellular N-CISSOR fragments was suppressed by pre- and co-treatment with GM6001, a broad-spectrum metalloprotease inhibitor, and uncleaved N-CISSOR accumulated in the cell lysate (Fig. 1d). The PMA-induced release of N-CISSOR ectodomain fragments increased in a time-dependent manner (Fig. 1e). These results were consistent with our results showing the PMA-induced cleavage of unlabelled zNRG1β1 (Supplementary Fig. S1a,b). The cleavage efficiency was comparable between N-CISSOR and unlabelled zNRG1β1 (Supplementary Fig. S1d). Moreover, the addition of culture medium from N-CISSOR-expressing HEK293T cells caused enhanced phosphorylation of ErbB3 and Akt in differentiated C2C12 cells (Fig. 1f), indicating that the cleaved N-CISSOR ECD possessed NRG1 bioactivity. We often found single-positive mCherry dots, without GFP signals, in N-CISSOR-expressing cells (Fig. 1g), which was not observed following treatment with Bafilomycin A1 (Supplementary Fig. S1f), suggesting that N-CISSOR passes through acidic compartments, similar to NRG1^9^ (described in detail in the Discussion section). Taken together, these data indicated that N-CISSOR behaved in a similar manner to NRG1.

### Live imaging of N-CISSOR ectodomain shedding in cultured cells

**Figure 2:**
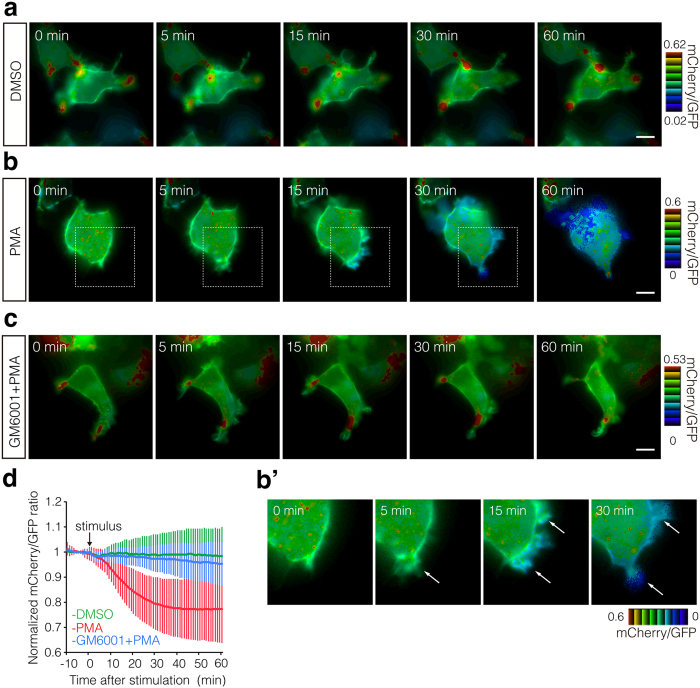
Real-time imaging of the PMA-induced ectodomain shedding of N-CISSOR. (**a–c**) Images of N-CISSOR-expressing, serum-starved HEK293T cells stimulated with DMSO (**a**), PMA (**b**), or a cocktail of GM6001 and PMA (**c**) were obtained every 1 min. In (**c**), cells were pre-treated with GM6001 for 30 min before stimulation. mCherry/GFP ratio images at the indicated time points are shown in the intensity-modulated display mode; 8 colours ranging from red to blue were used to represent the mCherry/GFP ratios of individual pixels, with the intensities indicating those of GFP. The upper and lower limits of the ratio range are shown on the right. Magnification of the cells shown in the white dotted box in (**b**) is shown in (**b’**). Arrows indicate the cellular protrusions. Scale bar: 10 μm. (**d**) Quantitative analysis of mCherry/GFP ratios in HEK293T cells transiently expressing N-CISSOR. mCherry/GFP ratios were normalized to the average mCherry/GFP ratio measured before stimulation. The mean normalized mCherry/GFP ratios and SD are shown (DMSO, n = 18; PMA, n = 15; GM6001 + PMA, n = 15). See also Supplementary Movie 1 and 2.

To demonstrate the utility of N-CISSOR as a probe for monitoring NRG1 ectodomain shedding, we imaged the dynamics of N-CISSOR in HEK293T cells by time-lapse imaging. HEK293T cells transiently expressing N-CISSOR were treated with PMA or dimethyl sulfoxide (DMSO; the solvent for PMA), and fluorescent microscopic images were obtained every 1 min for 1 h. PMA-treated cells showed a dramatic change in the mCherry/GFP ratio colour in a time-dependent manner after PMA treatment, whereas this colour ratio remained unchanged over time in control, DMSO-treated cells (Fig. 2a,b, Supplementary Movie 1), indicating the change in mCherry/GFP ratios was attributable to the release of mCherry-fused extracellular NRG1 moieties. Intriguingly, prominent and rapid changes in the mCherry/GFP ratios were often observed, especially in cellular protrusions, upon PMA treatment (Fig. 2b’, Supplementary Movie 2). The PMA-induced change in the mCherry/GFP ratio colour was clearly inhibited by pre- and co-treatment with GM6001, suggesting that the changes were dependent on metalloprotease activity (Fig. 2c). Quantitative analysis indeed indicated a distinct decrease of the normalized mCherry/GFP ratio after PMA stimulation (Fig. 2d).

### Development of a mutant N-CISSOR lacking NRG1 cleavage sites

**Figure 3:**
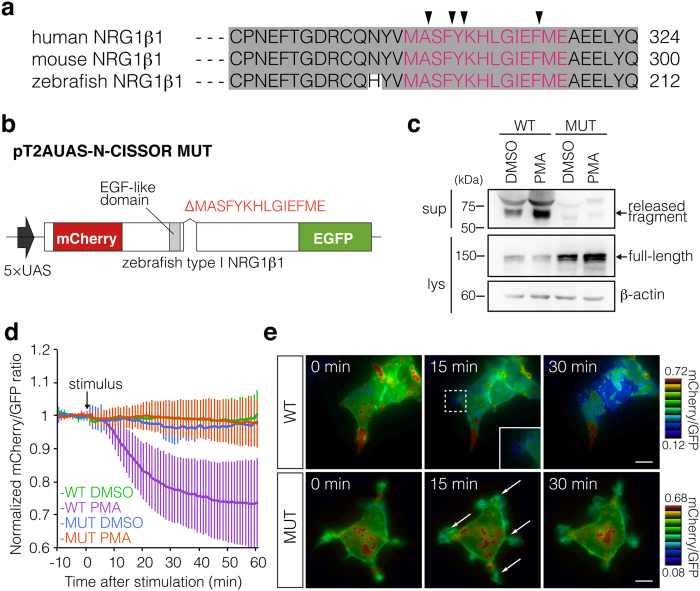
Dependence of N-CISSOR ectodomain shedding on the NRG1 cleavage sites. (**a**) Cross-species comparison of NRG1 amino acid sequences neighbouring the cleavage sites (arrowheads) among the human, mouse, and zebrafish proteins. (**b**) Structure of a mutant (MUT) N-CISSOR protein with a deletion of 14 amino acids (MASFYKHLGIEFME) neighbouring the cleavage sites. (**c**) Western blot analysis of N-CISSOR (WT/MUT) cleavage induced with DMSO or PMA for 20 min. (**d**) Quantitative analysis of mCherry/GFP ratios normalized to the average mCherry/GFP fluorescence ratio before stimulation. The mean normalized mCherry/GFP ratios and SD are shown (WT DMSO, n = 21; WT PMA, n = 16; MUT DMSO, n = 22; MUT PMA, n = 18). (**e**) Representative mCherry/GFP ratio images of PMA-stimulated WT or MUT N-CISSOR-expressing cells at the indicated time points are shown. Scale bar: 10 μm. See also Supplementary Fig. S2 and Supplementary Movie 3.

NRG1 type β can be cleaved at several sites within a short amino acid segment in its juxtamembrane domains encoded by β-exon located between the EGF-like and transmembrane domains^6^,^20^. Interestingly, the amino acid sequences including the reported cleavage sites of NRG1 are highly conserved among the human, mouse, and zebrafish (Fig. 3a), suggestive of a common mechanism of NRG1 processing. To confirm that N-CISSOR cleavage depends specifically on NRG1 cleavage sites, we generated a mutant N-CISSOR variant (N-CISSOR MUT, Fig. 3b) that lacked the responsible 14 amino acids, i.e., MASFYKHLGIEFME, which includes several cleavage sites. Western blot analysis showed impaired PMA-induced processing of N-CISSOR MUT in HEK293T cells. Accordingly, although N-CISSOR MUT could localize to the cell surface similarly to N-CISSOR (Supplementary Fig. S2a), no substantial decrease in the mCherry/GFP ratio was observed in response to PMA treatment (Fig. 3d, Supplementary Movie 3). N-CISSOR MUT also failed to show local changes in the mCherry/GFP ratio at protrusions in cells stimulated with PMA (Fig. 3e), showing that the mCherry/GFP colour ratio change of N-CISSOR in cellular protrusions was caused by the accurate shedding of NRG1 juxtamembrane domains encoded by the β-exon. Western blot analysis also showed that the attenuated processing of N-CISSOR MUT still took place (Fig. 3c), suggesting that NRG1 can be processed at sites other than removed 14 amino acids through minor or salvaged proteolytic pathway.

### *In vivo* visualization of N-CISSOR cleavage in neuronal cells

**Figure 4:**
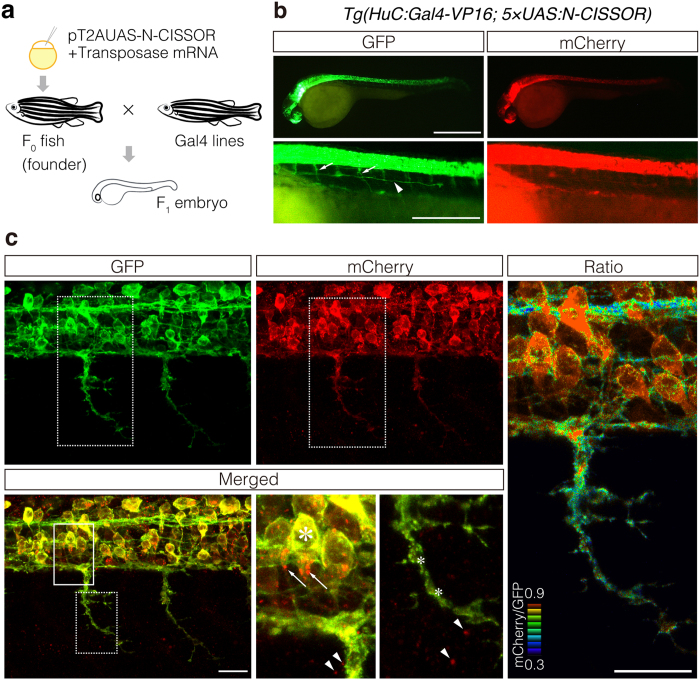
Expression of N-CISSOR in zebrafish. (**a**) Schematic representation of the procedure used for transgenesis and sample preparation. (**b,c**) Lateral view of the resulting F_1_ *Tg* (*HuC:Gal4-VP16; 5* *×* *UAS:N-CISSOR*) embryo at 36 hpf under a fluorescence stereomicroscope (**b**) and a confocal microscope (**c**), showing N-CISSOR expression in the nervous system. (**b**) Spinal motor nerves and posterior lateral line are indicated by the arrows and arrowhead, respectively. Scale bar: 200 μm. (**c**) Maximum projection of z-stack images and views of magnified areas surrounded by solid and dotted borders are shown. High colocalization of GFP and mCherry in somas (large asterisk) and low colocalization in axons (small asterisks). Intracellular (arrows) and extracellular (arrowheads) accumulation of mCherry. The mCherry/GFP fluorescence ratio image is shown in the right column. Scale bar: 20 μm.

To examine when and where NRG1 ectodomain shedding occurs *in vivo*, we generated transgenic zebrafish, which expressed N-CISSOR in a tissue-specific manner, using the Gal4-UAS system. F_0_ fishes, in which N-CISSOR cDNA was introduced, were crossed with transgenic fish, *Tg* (*HuC: Gal4-VP16*), expressing Gal4-VP16 in the nervous system, and their offspring (F_1_ embryos) were observed at 36 h post-fertilization (hpf; Fig. 4a,b). GFP fluorescence was clearly observed in the neural tissues, including the spinal motor nerves and posterior lateral lines (Fig. 4b). Photon-counting imaging under a confocal microscope enabled us to obtain high-resolution images of the N-CISSOR expressing spinal motor nerves in zebrafish embryos at 36 hpf. Although both mCherry and GFP were localized in the soma and axons, mCherry signals were weaker in the axons than those in the soma region where mCherry signals were strongly merged with GFP. Indeed, mCherry/GFP ratio images showed decreased ratios in the axons, compared to those observed in the somas. Punctate, single-positive mCherry fluorescence was observed intracellularly, as was observed in cultured cells. We also noted that the mCherry single-positive dots scattered predominantly in the extracellular regions of N-CISSOR-expressing neurons (Fig. 4c, arrowheads).

**Figure 5:**
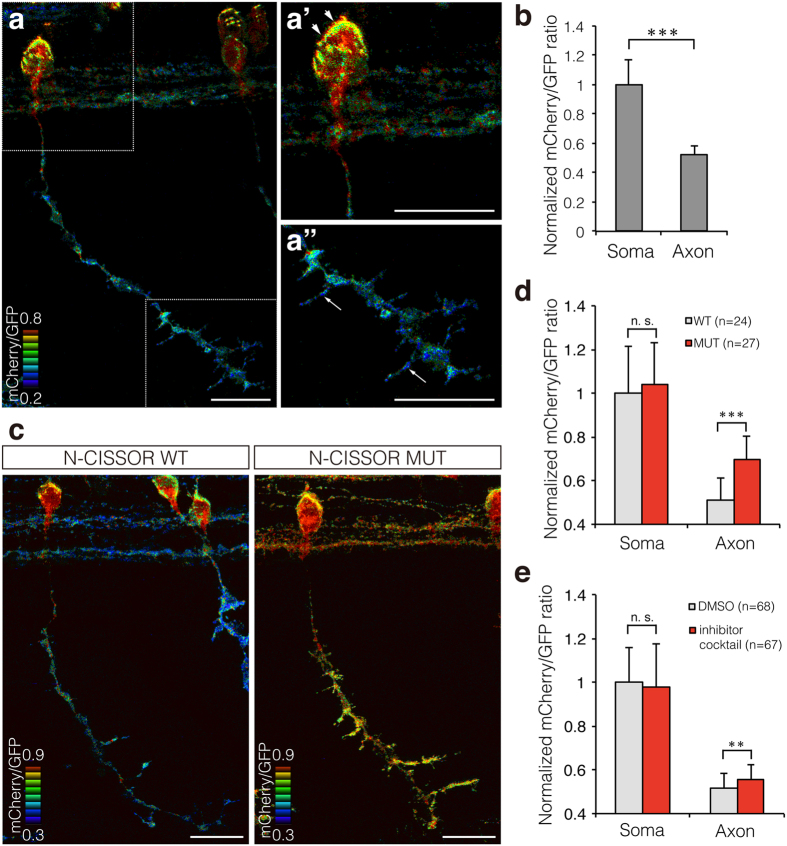
Single-cell analysis of N-CISSOR in zebrafish. (**a**) A representative mCherry/GFP ratio image of single CaP neurons in *Tg* (*SAIGFF213A; 5* × *UAS:N-CISSOR*) embryo at 36 hpf. (**a’**,**a”**) Magnified views of (**a**), showing higher ratio in the surface of soma (arrowheads) and axonal branches (arrows). Scale bar: 20 μm. (**b**) Comparison of mCherry/GFP fluorescence ratios in somas and axons of single or coupled CaP neurons. The mCherry/GFP ratios were calculated for GFP-positive regions and normalized to the mean mCherry/GFP ratio observed in somas. The data are shown as the mean ± SD. *P* values were calculated by performing Student’s t-test. ^***^P < 0.0001. (**c**) Representative mCherry/GFP images of N-CISSOR (WT or MUT)-expressing CaP neurons at 36 hpf. Scale bar: 20 μm. (**d**) Normalized mCherry/GFP ratios in GFP-positive regions analysed in somas and axons from *Tg* (*SAIGFF213A; 5* × *UAS-N-CISSOR*) and *Tg* (*SAIGFF213A; 5* × *UAS-N-CISSOR MUT*) embryos at 36 hpf. The data shown are the mean ± SD. *P* values were calculated by performing Student’s t-test. ^***^P < 0.0001. (**e**) Normalized mCherry/GFP ratios in GFP-positive regions analysed in somas and axons of single or coupled CaP neurons in *Tg* (*SAIGFF213A; 5* × *UAS-N-CISSOR*) treated with a protease inhibitor cocktail (GM6001 and BACE inhibitor IV) or vehicle control (DMSO), at 25–36 hpf. The data shown are the mean ± SD. *P* values were calculated by performing Student’s t-test. ^**^P < 0.001; ^***^P < 0.0001. See also Supplementary Fig. S3 and Supplementary Movie 4.

We analysed mCherry/GFP ratios in N-CISSOR-expressing cells at the single-cell level *in vivo*. To this end, we crossed *Tg* (*5* × *UAS:N-CISSOR*) with *Tg* (*SAIGFF213A*) expressing Gal4FF strongly in the CaP motor neurons. The hybrid Gal4 fish enabled observation of single or coupled CaP neurons and elongating axons toward individual myotomes. In the 36-hpf embryos, we reproducibly observed lower mCherry/GFP ratios in the axons than those in the somas of the CaP neurons (Fig. 5a, Supplementary Movie 4). In particular, branches of axons showed lower mCherry/GFP ratios (Fig. 5a’), indicating a higher shedding activity of N-CISSOR in the axons and axonal branches. Quantitative analysis revealed that the relative mCherry/GFP ratios were significantly lower in the axons than in the somas of CaP neurons (Fig. 5b). To determine whether the difference of mCherry/GFP ratios between the somas and axons was attributable to N-CISSOR cleavage, we also generated *Tg* (*5* × *UAS:N-CISSOR MUT*), which expressed N-CISSOR MUT (Fig. 3b) and crossed them with *Tg* (*SAIGFF213A*). In the CaP neurons of the resulting embryos, both mCherry and GFP accumulated at high levels in axons (Supplementary Fig. S3a), indicating that the transmembrane N-CISSOR protein translocated to the axons prior to cleavage. In addition, the axonal mCherry/GFP ratios of N-CISSOR MUT were significantly higher than those of N-CISSOR WT, while these ratios were comparable between the somas of these fishes. In this experiment, we observed higher mCherry/GFP ratios in the somas than those in the axons even in the N-CISSOR MUT-expressing fish. These higher values are partially due to preferential intracellular accumulation of single-positive mCherry fluorescences there. Moreover, we treated zebrafish embryos with a cocktail of protease inhibitors, GM6001, and BACE inhibitor IV, which efficiently inhibited N-CISSOR cleavage in mouse N1E115 neuroblastoma cells (Supplementary Fig. S3b). Although the inhibitor cocktail had no remarkable effect on the morphology of CaP neurons (Supplementary Fig. S3c), it caused a significant increase in the mCherry/GFP ratios in the axons, but not in the somas (Fig. 5e). These results suggested that the transmembrane NRG1 protein was preferentially processed in the axons rather than in the somas in developing motor neurons of zebrafish embryos.

## Discussion

In this study, we successfully developed a fluorescent probe, N-CISSOR, which enabled characterization of the subcellular localization and ectodomain shedding of transmembrane protein NRG1. Considering that correct NRG1 subcellular localization and/or protein modification is prerequisite for proper ectodomain shedding, we carefully designed N-CISSOR to mimic NRG1 behaviour as follows: First, the N-CISSOR protein included the ICD of NRG1 because phosphorylation of the ICD by PKCδ is essential for induced NRG1 cleavage^21^,^22^. Second, the probe included the transmembrane domain of NRG1 itself. The ectodomain shedding of NRG1 would be followed by γ-secretase-dependent intramembranous cleavage, and in some cases, subsequent nuclear translocation of the ICD might function as a transcriptional regulator^3^,^23^. Therefore, the transmembrane domain, where γ-secretase acts, is essential for the investigation of regulatory mechanisms of NRG1 ectodomain shedding. Finally, the entire N-CISSOR ECD was included so that it could interact with ErbB receptors because ligand-receptor interactions are likely required for NRG1 processing in some cases^23^, as in the case of Notch^24^, for which ectodomain shedding requires binding of its ligand Delta. Moreover, NRG1 passes through the lipid bilayer without a typical signal peptide on its N-terminus, suggesting that the existence of internal signal peptides within the ECD serve this role. Considering these factors, N-CISSOR was designed to include the entire zNRG1Iβ1 sequence.

This design differs from previously reported fluorescent probes used to monitor the proteolytic processing of transmembrane proteins; the previous probes excluded amino acid sequences other than cleavage sites^25^,^26^,^27^, or lacked functional domains such as ligand domains^28^. As expected, we confirmed the proper localization of N-CISSOR to the cell surface in HEK 293T cells, efficient ectodomain shedding upon PMA treatment similarly to unlabelled zNRG1I1 and HA-mNRG1Iβ1, and the ability to activate ErbB3 expressed in differentiated C2C12 myotubes. Taken together, these data indicated that N-CISSOR substantially mimics NRG1 in terms of its subcellular localization, processing, and bioactivity. The previously reported fluorescence probe for monitoring HB-EGF cleavage lacked of the EGF-like domain in order to minimize effects of overexpression^28^. On the other hand, we found that the deletion of EGF-like domain from N-CISSOR failed to mimic NRG1 cleavage dynamics induced by PMA; nevertheless it was translocated to cell surfaces as N-CISSOR (Supplementary Fig. S4a–c), suggesting that protein misfolding caused by excessive artificial protein modification resulted in ineffective cleavage by proteases or that EGF-like domain is indeed necessary for proper NRG1 cleavage. Therefore, we concluded that we should minimize the modification of transmembrane protein to mimic its cleavage dynamics.

Live cell imaging with N-CISSOR revealed a preferential shedding of N-CISSOR in the ruffling cellular protrusions of PMA-stimulated cells, showing that spatiotemporal regulation of NRG1 ectodomain shedding occurred. PMA induces ruffles and lamellipodia formation through actin reorganization in a Rac-dependent manner^29^. ADAM17, the main protease responsible for PMA-induced ectodomain shedding^30^, redistributes from the perinuclear region to the ruffling membrane through Rac-dependent actin reorganization in response to PMA treatment^31^,^32^,^33^. Therefore, it is plausible that PMA-induced dynamic reorganization of the actin cytoskeleton in cellular protrusions causes local ADAM17 activation, resulting in preferential N-CISSOR cleavage. In addition, PMA activates several PKCs. Interestingly, PMA-induced NRG1 cleavage requires the C-terminal phosphorylation of NRG1 by PKCδ, but not PKCα, whereas PMA-induced cleavage of TGFα and HB-EGF is regulated by PKCα^21^. Moreover, activated PKCδ is also translocated to the lamellipodia of spreading cells^34^. Taken together, these data suggest that preferential N-CISSOR cleavage in cellular protrusions might be attributed to, not only localized protease activity, but also the subcellular sensitization of NRG1 by PKCδ phosphorylation.

We employed N-CISSOR in transparent zebrafish embryos and observed greater NRG1 shedding in the axons than in the somas of motor neurons. Schwann cells migrate and myelinate along axons, but not along the cell bodies or dendrites of neurons. Previous studies suggested that Schwann cells secrete neurotrophic factors such as BDNF to locally induce Ig-NRG1 release from axons^16^,^17^. Therefore, axonal NRG1 release can be attributed to localized ectodomain shedding at neural axons. However, no previous data have shown localized NRG1 ectodomain shedding. In this study, by employing N-CISSOR in developing zebrafish, we directly demonstrated the preferential NRG1 ectodomain shedding at axons. Together with the preferential N-CISSOR shedding at the protrusions of PMA-stimulated cells, these findings reveal a significance of ectodomain shedding in the spatiotemporal regulation of NRG1 signalling, emphasizing the utility of N-CISSOR *in vivo*. In addition, the observation of increased axonal mCherry/GFP ratios following treatment with BACE and metalloprotease inhibitors implied that the NRG1 ectodomain in axons is regulated by BACE and/or metalloprotease activities. Insufficient effects of the protease inhibitors could be due to insufficient penetration of chemical inhibitors into embryos. Alternatively, proteases other than metalloproteases and BACEs might contribute to the leaky cleavages. Further studies are required to confirm the identity of the responsible proteases and to elucidate the mechanisms mediating the preferential shedding of axonal NRG1.

Although N-CISSOR effectively recapitulated NRG1 proteolytic processes, the need for improvement and further characterization remains. When N-CISSOR was expressed, mCherry single-positive dots were often found intracellularly, in spite of the absence of mCherry-tagged cleavage fragments in HEK293T cells (Supplementary Fig. S1c). Bafilomycin A1 treatment caused a distinct disappearance of mCherry single-positive dots and the appearance of mCherry and GFP double-positive membranous vacuoles that colocalized with late endosomal ECFP and mCFP-Rab7 (Supplementary Fig. S1e,f), suggesting that N-CISSOR is endocytosed and that subsequently, only GFP is quenched in low PH compartments. In agreement, a previous study suggested that NRG1 is trafficked through acidic compartments^9^. We also found mCherry single-positive dots residing outside of N-CISSOR-expressing motor neurons. Because the Ig-like domain of NRG1 binds to heparan sulphate proteoglycans and accumulates to sustain Ig-NRG1-ErbB-dependent signaling^35^, it is conceivable that the mCherry-single positive dots represented an accumulation of soluble N-CISSOR forms. Alternatively, those dots might be exosomes containing soluble forms of N-CISSOR or its full-length form, with quenched GFP being secreted from N-CISSOR-expressing neurons. Further characterization of these mCherry single-positive dots will enable better assessment of the subcellular activity of N-CISSOR processing, including that occurring in endosomal/lysosomal compartments where the GFP moiety of N-CISSOR is quenched by exposure to a low-pH environment by substitution with fluorescent proteins that are less sensitive to low pH.

The preferential cleavage of N-CISSOR in axons demonstrated in this study shows the potential of N-CISSOR for visualizing NRG1 cleavages. Further studies using N-CISSOR will elucidate regulatory mechanisms for axonal NRG1 shedding. Similarly, visualization of N-CISSOR in the heart will enable the assessment of how ectodomain shedding of NRG1 expressed in cardiac endothelial cells contributes to the trabeculation of myocardial cells during development, or how the cleavage of NRG1 in perivascular cells regulates heart regeneration^36^. In conclusion, using the N-CISSOR probe would enable new insights into the NRG1-mediated spatiotemporal regulation of cell signalling by monitoring NRG1 ectodomain shedding at subcellular resolution, both *in vitro* and *in vivo*. N-CISSOR would also be useful for the evaluation and screening of drugs that regulate NRG1 cleavage, not only in cultured cells, but also in living animals.

## Methods

### Chemical compounds

PMA, GM6001, and BACE inhibitor IV were purchased from Calbiochem (La Jolla, CA). Bafilomycin A1 was obtained from Sigma-Aldrich (St. Louis, MO, USA).

### Cell culture

HEK293T and C2C12 cells were purchased from the American Type Culture Collection (Manassas, VA, USA). Cells were maintained at 37 °C and 5% CO_2_ in DMEM culture medium (Thermo Fisher Scientific, Inc., Rockford, IL, USA), supplemented with 10% foetal bovine serum and 1% penicillin/streptomycin (P/S). For the differentiation of C2C12 cells, semi-confluent cells were incubated for 3 days in DMEM containing 2% horse serum.

### Animals

All animal experiments were approved by the ethics review boards for animal experiments of Kyoto University and were conducted in accordance with the Regulations on Animal Experimentation at Kyoto University. We sacrificed a minimal number of live animals, according to the institutional guidelines. Zebrafish were maintained in an aquarium at 28 °C under a light-dark cycle (14 h light, 10 h dark) and housed as described previously^37^.

### Plasmid construction

Full-length cDNA encoding the membrane-bound type-I form of zebrafish NRG1, β1-type (*zNRG1Iβ1*; DDBJ accession number LC126300) was cloned following RT-PCR amplification of RNA prepared from the whole brain of an adult zebrafish, using the following primers: forward: 5′-CTGCATCATGGCTGAGGTGAAAGC-3′ and reverse: 5′-GCATTTTCACACAGCTATAGGATC-3′. *mCherry* cDNA was inserted between nucleotides 89 and 90 of the *zNRG1Iβ1* open reading frame, and *EGFP* cDNA was added to the 3′ end of the gene (Fig. 1a). The fused *zNRG1Iβ1* (N-CISSOR) gene was cloned into the 5 × UAS sequences of the pT2AUASMCS vector^38^, which carries Tol2 elements (pT2AUAS-N-CISSOR). Mutant N-CISSOR (N-CISSOR MUT) was generated by inverse PCR, using the pT2AUAS-N-CISSOR vector (pT2AUAS-N-CISSORmut). To express these constructs in cultured cells using the Gal4-UAS system, Gal4-VP16 was inserted into the mammalian expression vector pEF-BOS (pEFBOS-Gal4-VP16). The full-length *zNRG1β1* gene was also inserted into the pEF-BOS vector (pEFBOS-zNRG1Iβ1). Human *RAB7A* cDNA was subcloned into the pCXN2-mCFP vector^39^ to generate the pCXN2-mCFP-Rab7 construct.

### Observation of N-CISSOR-expressing cells

HEK293T cells were plated in 35-mm glass-based dishes and transfected with the pT2AUAS-N-CISSOR or pT2AUAS-N-CISSORmut vector, together with pEFBOS-Gal4-VP16, pEFBOS-HA-mNRG1^40^, or pEFBOS-zNRG1Iβ1 using PEI-MAX (Polysciences Inc., Warrington, PA, USA). After an overnight incubation, the cells were fixed in 4% paraformaldehyde (PFA) in PBS for 5 min at room temperature and stained with DAPI, where necessary.

### *In vitro* time-lapse assay of N-CISSOR cleavage

HEK293T cells were plated in 35-mm glass-based dishes and co-transfected with the pEFBOS-Gal4-VP16 vector and either pT2AUAS-N-CISSOR or pT2AUAS-N-CISSORmut, using PEI-MAX. After an overnight incubation, the cells were starved for at least 1 h in serum-free Fluorobrite DMEM (Thermo Fisher Scientific) containing 1% P/S, 0.1% bovine serum albumin (BSA), and 1× GlutaMAX^TM^-I. Cells were treated with stimuli or inhibitors, if necessary. Time-lapse assays were performed with an Olympus IX81 inverted microscope, using a PlanApo 60x/1.40 oil objective lens, equipped as described previously^41^. Captured images were processed using MetaMorph software (Universal Imaging, West Chester, PA), as follows. After background subtraction, the efficiency of N-CISSOR cleavage was determined by analysing the mCherry/GFP fluorescence ratios in the intensity-modulated display mode; 8 colours, ranging from red to blue, were used to represent the mCherry/GFP ratio, with 32 colour grades indicating the signal intensities in the GFP images. For quantitative analysis, the mCherry and GFP intensities were averaged over the entire cell area, and the resulting data were exported to Excel software (Microsoft Corporation, Redmont, WA). The mCherry/GFP ratios were calculated and normalized to the average ratio measured at 10 min before chemical stimulation.

### Western blot analysis of N-CISSOR cleavage and bioactivity

HEK293T cells were plated in 60-mm dishes, transfected, and then starved in Opti-MEM for 1 h before chemical stimulation was applied. In some cases, cells were pre-treated with GM6001 (50 μM) for 30 min before PMA stimulation. The supernatants of HEK293T cells transiently expressing N-CISSOR were collected after stimulation and concentrated by ultrafiltration. The cells were washed with cold PBS and lysed in extract buffer^40^. Protein concentrations were determined using the Pierce^TM^ Protein Assay Kit (Life Technologies, Carlsbad, CA). The resulting samples were denatured in sodium dodecyl sulphate buffer containing 10% 2-ME for 5 min at 95 °C. The same quantity of protein in lysates or sample supernatants (15–20 μg/lane) was loaded and separated on 10% polyacrylamide gels or on 4–20% pre-cast polyacrylamide gels (Bio Rad, München, Germany), after which the proteins were transferred to polyvinylidene difluoride membranes (Immobilon-P; Millipore, Billerica, MA). After blocking with 1 or 5% skim milk, the membranes were incubated for 24 h at 4 °C with primary antibodies, including an anti-mCherry antibody (clone 1C51, Abcam, Cambridge, MA; 1:2,000), an anti-GFP antibody (clone AB3080, Millipore, Germany; 1:2,000), or an anti-β-actin antibody (clone AC-74, Sigma Aldrich, Saint Louis, MO; 1:4,000). The cleavage of zNRG1Iβ1 was assessed similarly using rabbit antibodies against the N terminus (683) or C-terminus (686) of zebrafish NRG1Iβ1 (See also Supplemental Information). For bioactivity assays, differentiated C2C12 cells plated in 60-mm dish were stimulated with conditioned medium from transfected HEK293T cells for 30 min. Human recombinant NRG (10 nM; R&D Systems, Inc., Minneapolis, MN, USA) and a vehicle control (0.1% BSA in PBS) were incubated with differentiated C2C12 cells as positive and negative control, respectively. Western blot was performed using 1:1,000-diluted antibodies against phospho-ErbB3 (clone 21D3,) or phospho-Akt (clone C31E5E), which were obtained from Cell Signalling Technology, Inc. (Danvers, MA, USA).

### Transgenesis in fish and observations

The pT2AUAS-N-CISSOR/N-CISSOR MUT vector was co-injected with Tol2 transposase mRNA into fertilized eggs. The injected fish was crossed with *Tg* (*HuC:Gal4-VP16*) fishes^42^ or *Tg* (*SAIGFF213A*) fishes^43^. The resulting F_1_ embryos were raised in the presence of 0.2 mM PTU (Wako *P*ure Chemical Industries, Ltd., Osaka, Japan) to block pigmentation. Embryos at 36 hpf were fixed in 4% PFA/PBS, immersed serially in PBS, 30% glycerol/PBS, and 50% glycerol/PBS and then observed under a Leica M205 C fluorescent stereomicroscope. For high-resolution imaging or ratio imaging, images were acquired in photon-counting mode using a Leica TCS SP8 confocal microscope equipped with the hybrid detector Leica HyD, using HC PL APO CS2 20×/NA 0.75, 40×/NA 1.30, and 63×/NA 1.40 objectives. Maximum-intensity projection of z-stack images was created with LAS AF software (Leica MicroSystems, Manheim, Germany) and used to generate ratio images and for quantification. Ratio images were generated in the same way as described above for cell culture analysis. The brightness and contrast were adjusted with MetaMorph software. For the quantitative analysis of mCherry/GFP ratios, following median filtering, GFP-positive regions were determined by voluntary thresholding, and the mCherry intensities were divided by GFP intensities in GFP-positive regions. The average mCherry/GFP ratios were measured in both the cell-body areas and the axonal areas. The axonal lengths were traced and measured using MetaMorph software. Single or coupled N-CISSOR-positive neurons with axons >80 μm were used for analysis.

### Inhibitor treatment of fish

An inhibitor cocktail was used at a final concentration of 0.5 mM GM6001 and 0.1 mM BACE inhibitor IV in 2% DMSO/embryo medium. Control embryos were treated with 2% DMSO/embryo medium. *Tg* (*SAIGFF213A; 5* × *UAS:N-CISSOR*) F_1_ embryos were dechorionated and raised in the presence of the inhibitor cocktail or 2% DMSO at 25–36 hpf, followed by fixation with 4% PFA/PBS at 36 hpf. The resulting fish were analysed as described above.

### Statistical analysis

The data were evaluated with two-tailed independent Student’s t-test using Microsoft Excel software. Probability values (P values) < 0.05 were considered statistically significant (^**^P < 0.01; ^***^P < 0.001). The data are presented in terms of the mean ± SD.

## Additional Information

**How to cite this article**: Kamezaki, A. *et al*. Visualization of Neuregulin 1 ectodomain shedding reveals its local processing *in vitro* and *in vivo*. *Sci. Rep*. **6**, 28873; doi: 10.1038/srep28873 (2016).

## Supplementary Material

Supplementary Information

Supplementary Movie S1

Supplementary Movie S2

Supplementary Movie S3

Supplementary Movie S4
