# Corrigendum: Visualization of Neuregulin 1 ectodomain shedding reveals its local processing *in vitro* and *in vivo*

**DOI:** 10.1038/srep46009

**Published:** 2017-04-20

**Authors:** Aosa Kamezaki, Fuminori Sato, Kazuhiro Aoki, Kazuhide Asakawa, Koichi Kawakami, Fumio Matsuzaki, Atsuko Sehara-Fujisawa

Scientific Reports 6: Article number: 2887310.1038/srep28873; published online: 07 01 2016; updated: 04 20 2017

The Acknowledgements section in this Article is incomplete.

“We are grateful to Drs K. Shimamura, J. Hatakeyama, M. Suzuki, and K. Tsumagari for helpful discussions. We also thank Drs Y. Kanaho and S. Higashijima for providing the murine neuroblastoma cell line N1E-115 and the *Tg (HuC:Gal4*-*VP16*) zebrafish, respectively. We thank Z. Wang for conceiving of the name N-CISSOR. This work was supported by MEXT KAKENHI Grant Number 22122007 (to A. Sehara-Fujisawa) and 15H02370 (to K. Kawakami); JSPS KAKENHI Grant Numbers 26650077 (to A. Sehara-Fujisawa) and 14J05637 (to A. Kamezaki); the National BioResource Project (to K. Kawakami); the Cooperative Research Program of Institute for Frontier Medical Sciences of Kyoto University (to K. Kawakami); and the Platform for Dynamic Approaches to Living System from the Ministry of Education, Culture, Sports, Science and Technology, Japan (to A. Sehara-Fujisawa and K. Aoki)”.

should read:

“We are grateful to Drs K. Shimamura, J. Hatakeyama, M. Suzuki, and K. Tsumagari for helpful discussions. We also thank Drs Y. Kanaho and S. Higashijima for providing the murine neuroblastoma cell line N1E-115 and the Tg (*HuC:Gal4*-*VP16*) zebrafish, respectively. We thank Z. Wang for conceiving of the name N-CISSOR. This work was supported by MEXT KAKENHI Grant-in-Aid for Scientific Research on Innovative Areas 22122007 and 15H05938 (to A. Sehara-Fujisawa) and 15H02370 (to K. Kawakami); JSPS KAKENHI Grant Numbers 26650077 and (B) 16H04793 (to A. Sehara-Fujisawa) and 14J05637 (to A. Kamezaki); the National BioResource Project (to K. Kawakami); the Cooperative Research Program of Institute for Frontier Medical Sciences of Kyoto University (to K. Kawakami); and the Platform for Dynamic Approaches to Living System from the Ministry of Education, Culture, Sports, Science and Technology, Japan (to A. Sehara-Fujisawa and K. Aoki)”.

