# Supplementary material for: Visualization of Neuregulin 1 ectodomain shedding reveals its local processing in vitro and in vivo
Source: Sci Rep. 2016 Jul 1;6:28873. doi: 10.1038/srep28873 (PMC4929465; doi:10.1038/srep28873)
Supplement: Supplementary Information [file srep28873-s1.pdf]

Supplementary Information for

Visualization of Neuregulin 1 ectodomain shedding reveals its local processing *in vitro* and *in vivo*

Aosa Kamezaki[1], Fuminori Sato[2], Kazuhiro Aoki[3], Kazuhide Asakawa[4], Koichi Kawakami[4], Fumio Matsuzaki[5], Atsuko Sehara-Fujisawa[2]*

[1]Department of Animal Development and Physiology, Graduate School of Biostudies, Kyoto University, Kyoto 606-8501, Japan

[2]Department of Growth Regulation, Institute for Frontier Medical Sciences, Kyoto University, Kyoto 606-8507, Japan

[3]Imaging Platform for Spatio-Temporal Information, Graduate School of Medicine, Kyoto University, Kyoto 606-8501, Japan

[4]Division of Molecular and Developmental Biology, National Institute of Genetics, and Department of Genetics, SOKENDAI, The Graduate University for Advanced Studies, Mishima, Shizuoka 411-8540, Japan

[5]Laboratory of Cell Asymmetry, RIKEN Center of Developmental Biology, Kobe 650-0047, Japan

*Author for correspondence (asehara@frontier.kyoto-u.ac.jp)

Supplementary Fig. S1

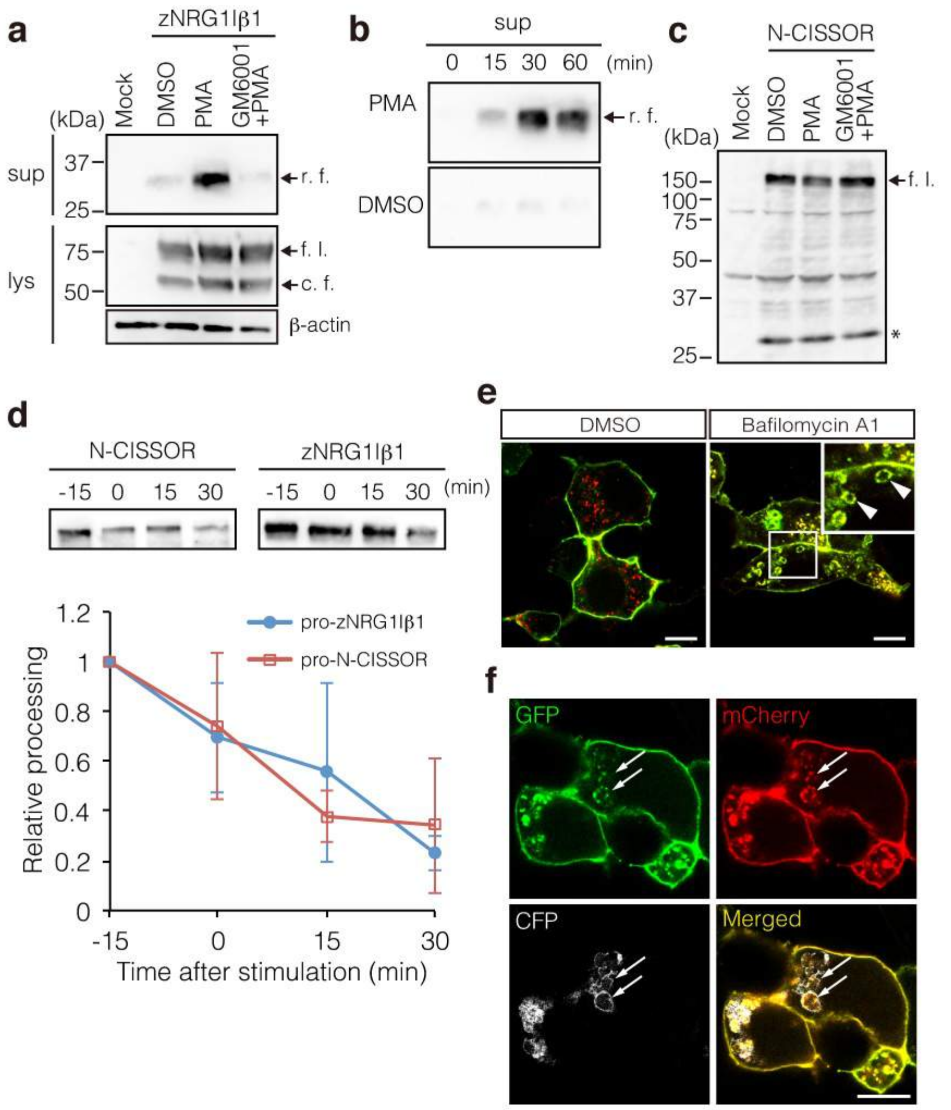

Supplementary Figure S1. Characterization of zNRG1Iβ1 and N-CISSOR cleavage. (**a–b**) Western blot analysis of (**a**) time- and (**b**) metalloprotease-dependent cleavage of zNRG1Iβ1 in HEK293T cells stimulated with PMA. Released fragments (r. f.: approximately 37 kDa) were detected in the supernatant using an anti-N terminus zNRG1I antibody (683). Full-length (f. l.: approximately 75 kDa) and cleaved fragments (c. f.: greater than 50 kDa) were detected in cell lysates, which were detected using an anti-C-terminus zNRG1 antibody (686), are shown. The supernatants were collected at the indicated time points. (**c**) Western blot analysis of mCherry-tagged fragments in the cell lysates of N-CISSOR-expressing HEK293T cells, using an anti-mCherry antibody. The asterisk shows extra bands produced by the intramolecular cleavage of mCherry during

the denaturation procedure. (**d**) Comparison of cleavage efficiency between zNRG1Iβ1 and N-CISSOR by Western blotting using 686 antibody. The means of relative intensities which were normalized with the β-actin intensity of each lane are shown as relative processing (zNRG1Iβ1: n=3, N-CISSOR: n=3). The data shown are the mean ± SD. (**e–f**) Bafilomycin A1 treatment of HEK293T cells expressing N-CISSOR alone (**e**) or co-expressing N-CISSOR and mCFP-Rab7 (**f**). Arrowheads indicate membranous signals merged with mCFP-Rab7 Scale bar: 10 μm

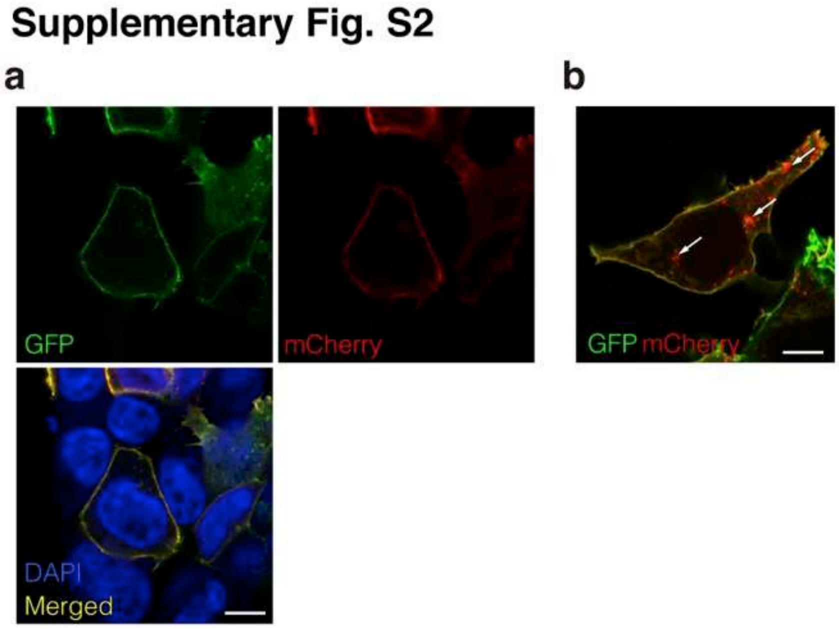

Supplementary Figure S2. Subcellular distribution of N-CISSOR MUT in HEK293T cells. (**a–b**) A N-CISSOR MUT-expressing HEK293T cells with (**a**) or without (**b**) DAPI staining. Arrows indicate the intracellular accumulation of mCherry single-positive signals. Scale bar: 10 μm

Supplementary Fig. S3

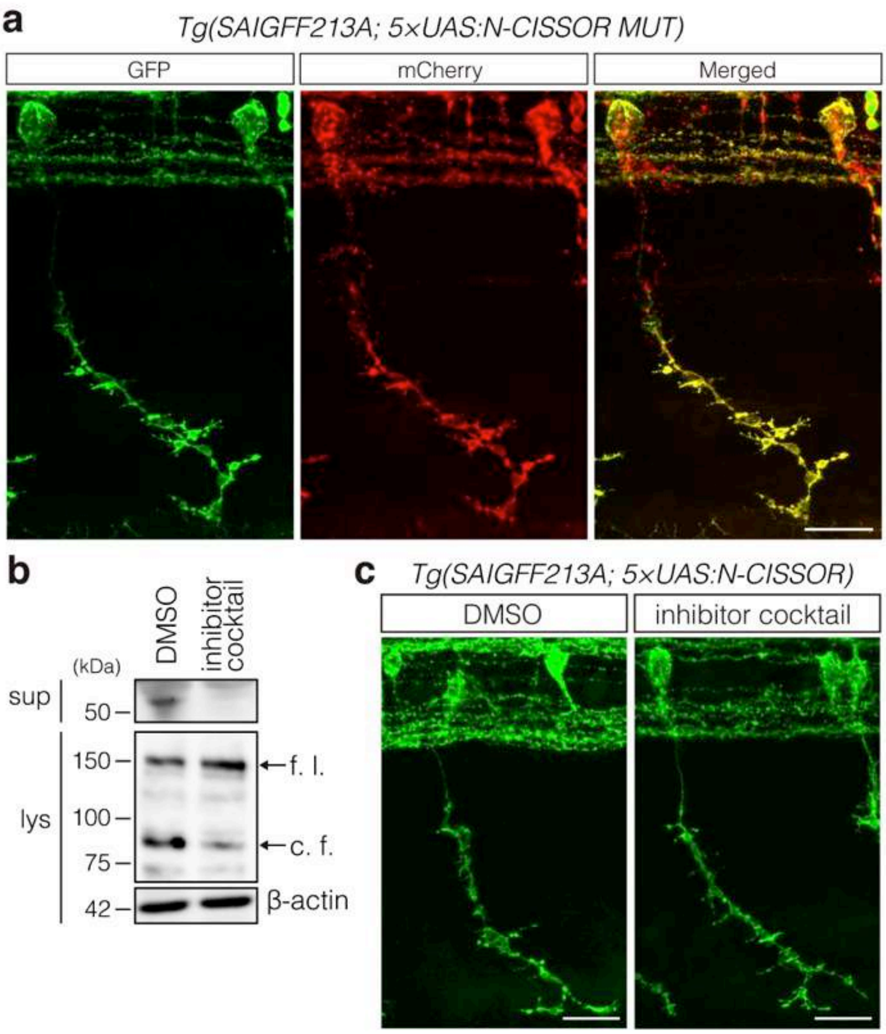

Supplementary Figure S3. Inhibition of N-CISSOR cleavage in zebrafish embryos. (**a**) Representative image of *Tg (SAIGFF213A; 5×UAS-N-CISSOR MUT)* at 36 hpf. Scale bar: 10 μm. (**b**) Western blot analysis of the inhibition of N-CISSOR cleavage following treatment with an inhibitor cocktail (0.5 μM GM6001 and 0.1 μm BACE inhibitor IV; dissolved in DMSO) for 4 h. f. l.: full-length; c. f.: cleaved fragment. (**c**) GFP image of CaP neurons in *Tg (SAIGFF213A; 5×UAS-N-CISSOR)* treated with an inhibitor cocktail (0.5 mM GM6001 and 0.1 mM BACE inhibitor IV; dissolved in DMSO) obtained between 25 and 36 hpf. Scale bar: 20 μm

Supplementary Fig. S4

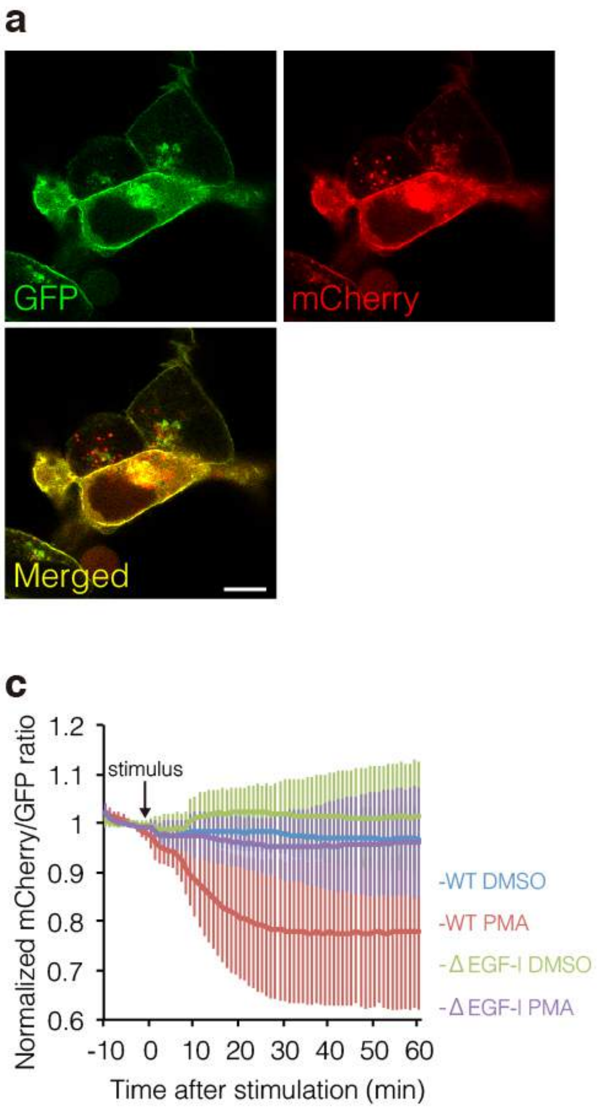

Supplementary Figure S4. Deletion of EGF-like domain from N-CISSOR.

(**a**) HEK293T cells which express N-CISSOR lacking of EGF-like domain (N-CISSOR Δ EGF-l). Scale bar: 10 μm (**b**) Western blotting analysis of N-CISSOR and N-CISSOR Δ EGF-l cleavage following treatment with PMA for 20 min. (c) Quantitative analysis of mCherry/GFP ratios in HEK293T cells transiently expressing N-CISSOR/N-CISSOR Δ EGF-l. mCherry/GFP ratios were normalized to the average mCherry/GFP ratio measured before stimulation. The mean normalized mCherry/GFP ratios and SD are shown (N-CISSOR DMSO: n=22, N-CISSOR PMA: n=22, Δ EGF-l DMSO: n=15, Δ EGF-l PMA: n=20).

Supplementary Movies

Supplementary Movie 1. Live-cell mCherry/GFP ratio imaging of N-CISSOR-expressing HEK293T cells. The cells were stimulated with DMSO (left), PMA (middle), or GM6001 and PMA (right) (related to Fig. 2)

Supplementary Movie 2. Preferential mCherry/GFP ratio decreases in the cellular protrusions. Enlarged images of PMA-stimulated ruffling cells in Movie S1. The arrow and the arrowhead indicate the formation of protrusions (related to Fig. 2)

Supplementary Movie 3. Comparison of mCherry/GFP ratio changes in N-CISSOR (WT) and N-CISSOR (MUT)-expression cells. Live-cell mCherry/GFP ratio imaging of N-CISSOR (WT)- or N-CISSOR MUT-expressing cells stimulated with PMA (related to Fig. 3)

Supplementary Movie 4. mCherry/GFP ratio images of a CaP motor neuron. A series of z-stack images of an N-CISSOR-expressing CaP motor neuron in Fig. 5a is shown as the mCherry/GFP ratio image.

Methods

Antibodies

A rabbit antibody (683) was produced against the KESVRSNKPDNSPQAEPKLC peptide, which is present in the N-terminal domain of zebrafish type-I NRG1. Another rabbit antibody (686) was produced against the CLEGSRTNPALHLSPQHE peptide, which is present in the C-terminal domain of the zebrafish NRG1 protein. These antibodies were used at a 1:1,000 dilution for western blot analysis.

Treatment of N-CISSOR-expressing HEK293T cells with bafilomycin A1

HEK293T cells were co-transfected with the pT2AUAS-N-CISSOR and pEFBOS-Gal4-VP16 vectors, with or without the pCXN2-mCFP-Rab7 vector. 4 h after transfection, the cells were treated overnight with 50 nM bafilomycin A1. The cells were washed, fixed with 4% PFA/PBS, and observed under a Leica SP8 confocal microscope.

Treatment of N-CISSOR-expressing N1E-115 cells with a protease inhibitor cocktail

N1E-115 cells were co-transfected with the pT2AUAS-N-CISSOR and pEFBOS-Gal4-VP16 vectors. After transfection, the medium was changed to Opti-MEM containing an inhibitor cocktail (50 μM GM6001 and 10 μM BACE inhibitor IV) and incubated for 4 h. The cellular supernatant and lysate were harvested, after which western blotting was performed, as described in the Methods section of the manuscript.

Comparison of cleavage efficiency between N-CISSOR and unlabelled zNRG1Iβ1

HEK293T cells were co-transfected with the pT2AUAS-N-CISSOR and pEFBOS-Gal4-VP16 vectors, or transfected with pEFBOS-zNRG1Iβ1. After over night incubation, the cells were starved for 1 h with Opti-MEM prior to PMA stimulation. The cellular lysate of the stimulated cells were harvested, after which western blotting was performed, as described in the Methods section of the manuscript. The signal intensities of full-length bands were measured using MetaMorph software after background subtraction and normalized with the intensity of β-actin . The normalized intensities were shown as relative value to the maximum intensity.

Generation of N-CISSOR lacking of EGF-like domain

N-CISSOR ΔEGF-l was generated by inverse PCR to remove the amino acid sequences, PCNESEKEYCVNHGKCFTLEVTPGNIRR.
